# Effects of Alcohol Use and Estrogen on Bone

**Published:** 2001

**Authors:** Russell T. Turner, Jean D. Sibonga

**Affiliations:** Russell T. Turner, Ph.D., is a consultant in the Department of Orthopedics, Mayo Clinic, professor of Orthopedics, and associate professor of Biochemistry and Molecular Biology at the Mayo Medical School, Rochester, Minnesota. Jean D. Sibonga, Ph.D., is an assistant professor in the Department of Orthopedics, Mayo Clinic, Rochester, Minnesota

**Keywords:** chronic AODE (effects of alcohol or other drug use, abuse, and dependence), estrogens, osteoporosis, risk factors, bone fracture, bone resorption, bone mass density, beneficial vs. adverse drug effect, biological repair, post menopause, gender differences

## Abstract

In marked contrast with men who drink, women who drink alcohol are found, as a group, to have higher bone mass compared with women who abstain. Furthermore, the apparent beneficial effects of alcohol use are more apparent in postmenopausal women than women of reproductive age, suggesting that there might be an interaction between alcohol and estrogen. Estrogen deficiency accompanying menopause leads to bone loss, which in turn predisposes women to osteoporosis later in life. Estrogen deficiency accelerates bone remodeling, which is the process by which small areas of bone are destroyed and rebuilt, and leads to an imbalance whereby bone resorption—the part of remodeling consisting of breaking down and assimilating—exceeds bone formation. Alcohol might reduce bone loss in postmenopausal women by increasing the circulating levels of estrogen. Alternatively, alcohol might slow bone loss by acting on bone cells to reduce bone remodeling. Alcohol use has a negative effect on the immature skeleton but current understanding suggests that small quantities of alcohol may have beneficial effects on bone in older women.

Bone is a living tissue that undergoes lifelong remodeling (Frost 1969), whereby local regions of the bone are destroyed and rebuilt in a systematic way (see figure). This process serves to repair microdamage caused by normal body wear and tear and is essential to maintaining strong bones. Bone is lost during the normal aging process. Osteoporosis occurs when a person has an inadequate amount of bone to provide sufficient strength to perform normal daily activities. Osteoporosis usually is caused by a chronic imbalance in the bone remodeling cycle in which bone resorption (shown in part B of the figure) is not adequately compensated for by subsequent bone formation (shown in part D of the figure).

Bone mass is not constant; it reaches a peak value in the third decade of life and then declines with age. A low peak bone mass and rapid age-related loss in bone mass predispose a person to developing osteoporosis. The onset of bone loss during middle age can precede an increased risk of fractures by as much as 2 decades, during which period the person rarely shows outward signs of any problem (i.e., is asymptomatic). For example, a woman may start to lose bone mass at age 45, experience a fracture at age 65, but have no symptoms of the bone loss during the 20 years in between. No methods currently exist to restore large amounts of bone to a depleted skeleton, although hormones that stimulate bone formation are being investigated for this purpose. Therefore, reducing the rate of bone loss that occurs with aging is the only effective way to reduce osteoporotic fractures. Two important controllable risk factors for osteoporosis are a deficiency in the hormone estrogen and heavy alcohol use.^1^ This article will focus on these two factors by discussing estrogen and alcohol’s effects on bone remodeling and the possible influence of alcohol on the bones of estrogen-deficient women.

## Medical and Public Health Costs of Musculoskeletal Disorders

The true extent of musculoskeletal disorders in the general population is not widely appreciated. Musculoskeletal complaints like arthritis, back pain, and bone fractures are among the most common reasons why patients see physicians and are hospitalized. The annual direct and indirect costs of musculoskeletal disorders in the United States exceed $125 billion (Praemer et al. 1992). Direct treatment costs make up a little less than half of the economic burden; morbidity, mortality, and the value of lost productivity account for most of the remaining costs.

There are more than 6 million bone fractures in the United States per year, and roughly 5 percent of fractures do not heal properly and require additional and often costly medical care. Only one-sixth of the fracture total is a result of osteoporosis. However, the consequences of osteoporotic fractures can be disproportionately severe. Hip fractures, for example, are especially dangerous, and two-thirds of the more than 275,000 hip fractures that occur annually in the United States are a result of osteoporosis. The common perception that a fracture is not life threatening is incorrect. One-fifth of patients with hip fractures die within 6 months of having the fracture, and those who survive generally have a poor prognosis for complete recovery. Of the survivors, one-fifth will require long institutionalization and another fifth face a permanent disability, often with mobility limited to a walker or wheelchair (Cooper and Melton 1996).

The prevalence of osteoporosis and the number of hip fractures are increasing at an alarming rate. For example, a recent estimate of the number of hip fractures in women worldwide was 1.2 million. This number is expected to grow to 4.5 million by the year 2050. The second half of the last century witnessed an unprecedented increase in lifespan. Longer life combined with the temporary increase in birth rate in the years immediately following the Second World War will greatly increase the number of Americans at risk for osteoporosis in the next 2 decades (Cooper and Melton 1996).

## Estrogen Deficiency as a Risk Factor for Osteoporosis

Estrogen influences virtually all aspects of bone physiology throughout life (Turner et al. 1994) The hormone plays an important role in maintaining bone mass in adult women, in part by slowing bone remodeling and in part by maintaining the proper balance between the activity of bone-forming cells (i.e., osteoblasts) and bone-resorbing cells (i.e., osteoclasts). The effects of estrogen and estrogen deficiency on bone remodeling are summarized in the table. When estrogen is deficient, there is an increase in the activation of new bone remodeling units. A remodeling unit is the discrete region on a bone surface where the remodeling sequence (shown in the figure) occurs. Increased activation frequency can result in an increase in fracture risk because the resorption and formation phases of the bone-remodeling cycle occur weeks or months apart. As shown in part B of the figure, resorption of bone by osteoclasts creates a cavity on the bone surface. This local thinning creates a “weak link” in the network of tissue that makes up the bone’s structure (i.e., the trabecular network). Accumulating many weak links in a small area could make the bone susceptible to fracture. The greater the remodeling rate, the greater the number of weak links, and the greater the likelihood that the bone will fracture.

In addition to this immediate degradation of bone strength, estrogen deficiency also affects bone-remodeling balance. Estrogen reduces osteoclast lifespan and lengthens the lifespan of the bone-forming cells. In estrogen-deficient women, osteoclasts are believed to excavate deeper resorption cavities, which osteoblasts are unable to refill completely. This leads to a negative remodeling balance in which there is a small amount of bone lost at every location where bone has undergone remodeling. The combination of increased bone-remodeling units and a negative remodeling balance is the basis for the rapid decrease in bone mass that follows menopause (Turner et al. 1994).

Once bone mass has declined below a critical threshold, fractures may occur as a result of normal daily activities. When bone mass falls to this level, with or without an existing fracture, the patient is diagnosed as having osteoporosis. This critical threshold is approximately at the lower limit of the normal range for premenopausal women. As bone loss continues, the risk for fractures increases.

Both the spongelike cancellous bone located in the marrow cavity and the more compact cortical bone forming the shaft are at risk of bone loss*.* Over a lifetime, women lose about 50 percent of their peak cancellous bone and about 35 percent of their peak cortical bone. Excessive bone loss resulting from estrogen deficiency is believed to be the most important of the many factors that determine the overall risk for osteoporosis in women (Richelson et al. 1984).

Menopause occurs in women as they progress through middle age. Osteoporosis, however, is not inevitable. Thus, it is clear that other factors influence whether a person develops osteoporosis*.* Genetics play an important role. In general, African Americans are at a lower risk for osteoporosis than Americans of European ancestry. In addition, lifestyle choices play an important role in maintaining skeletal health. For example, adequate calcium intake and exercise reduce osteoporosis risk whereas smoking and heavy drinking increase risk.

## Alcohol Use as a Risk Factor for Osteoporosis

Overwhelming evidence from human and laboratory animal studies shows that chronic heavy drinking has detrimental effects on the skeleton (Turner 2000). The development of osteoporosis in middle-aged men is uncommon in the general population but is associated with alcoholism among men. Decreases in bone mass in male alcoholics have been documented by X-rays, measures of bone density, and by microscopic study of body tissues (i.e., histology). As reported in a recent review (Turner 2000), histological studies have shown that, compared with people in good health, male alcoholics have reductions in the total amount of cancellous bone as well as in the thickness of individual bone struts (bone support structures). Research (primarily in laboratory animals) shows that alcohol use also results in the production of bone with reduced strength (Hogan et al. 1997). As a consequence, bone strength in alcoholics of both genders may be diminished even more than predicted from bone mass measurements.

No studies have examined the effects of alcohol use during childhood and adolescence on bone remodeling in humans, but extensive research in this area has been conducted with growing rats (Sampson et al. 1997). Extrapolation from studies conducted in laboratory animals strongly suggests that frequent drinking before the skeleton is fully mature will reduce peak bone mass and, as a consequence, will result in an immediate increase in fracture risk and predispose the skeleton to osteoporotic fractures later in life.

## Gender Differences in the Skeletal Response to Alcohol

Because most studies of the effects of alcohol on bone health have been performed in men, researchers have not been able to estimate reliably the effects of alcohol use on women’s bones. In contrast to the findings for men, however, little evidence exists of bone loss in female heavy drinkers. Indeed, heavy alcohol use has been associated with an increased fracture rate in men but not in women (see Turner 2000).

The effects of moderate alcohol consumption on bone health are relevant to a greater number of people, compared with the effects of heavy alcohol consumption, but are even less certain. Reports of improved bone mass being associated with moderate alcohol use, especially among postmenopausal women, are intriguing (Laitinen et al. 1993). However, studies in rats indicate that bone formation is suppressed when alcohol is consumed at a level comparable to light-to-moderate consumption in humans (Turner 2000). Such findings emphasize the need for more definitive studies of the effects of light and moderate alcohol consumption.

## Action of Alcohol on Bone Growth and Remodeling

The effects of alcohol use on bone remodeling are summarized in the table. Histological analysis in male alcoholics has shown that chronic heavy drinking is associated with decreased osteoblast activity (see Turner 2000). These findings are consistent with studies that show an association between heavy alcohol use and reduced levels of osteocalcin, a biochemical marker of bone formation (Turner 2000). In contrast, histological and biochemical markers of bone resorption do not appear to be consistently affected by alcohol use. However, dose response studies in adult rats (i.e., studies that evaluate the effects of varying alcohol doses) have shown that moderate alcohol consumption decreased both bone formation and bone resorption (Turner 2000). Heavy alcohol consumption resulted in further decreases in bone formation with no additional decrease in bone resorption, resulting in bone loss (Turner et al. 2001). Although the effects of varying amounts of alcohol on the human skeleton have not been measured directly, there is evidence that only moderate amounts of alcohol are required to reduce blood levels of osteocalcin, suggesting that alcohol use results in a similar decrease in bone remodeling in humans (Nielsen et al. 1990).

The observed changes in osteoclast and osteoblast numbers in people who drink alcohol suggest a wide range of alcohol consumption levels may reduce the rate of initiation of bone remodeling sites*.* In addition, heavy alcohol consumption may cause an imbalance between bone formation and bone resorption. Alcohol is known to interfere in the expression of proteins that mediate bone remodeling and in the balancing of bone formation to bone resorption, suggesting a possible mechanism for alcohol’s effects (Turner et al. 1998). In support of this hypothesis, alcohol has been shown to reduce the expression of insulin-like growth factor-1, an important survival factor for osteoblasts in bone (Turner et al. 1998).

On the other hand, not all alcoholics exhibit low bone mass. Furthermore, it has been difficult to demonstrate either alcohol-induced bone loss or increased fracture rates in studies of both genders (Turner 2000). Indeed, most studies of women have shown a positive association between alcohol consumption and bone mass and no change or a decrease in fracture risk (Turner 2000). Overall, the evidence generally suggests that chronic heavy drinking has a detrimental skeletal effect for a subpopulation of men and that moderate alcohol consumption has a neutral or generally beneficial skeletal effect, especially in postmenopausal women (Turner 2000). This variability in the skeletal response to alcohol strongly implicates an important role for more multiple risk factors, such as genetic susceptibility and other lifestyle factors (e.g., smoking).

The markedly different effects of alcohol use on bone mass in young men and women compared with post-menopausal women may be understood by considering the effects of alcohol on bone growth and remodeling. An inhibitory effect of alcohol on bone growth in adolescent boys and girls would lead to a reduction in peak bone mass, a state that predisposes one to bone fractures later in life. An imbalance between bone formation and resorption in young adult male and female alcohol abusers, particularly when resorption predominates, would lead to gradual bone loss despite decreased bone remodeling. This is in sharp contrast to the decrease in bone remodeling in postmenopausal women who consume alcohol, which slows bone loss relative to their peers who abstain.

## Alcohol and Estrogen Interactions

Animal research has demonstrated the effects of estrogen on bone remodeling (see sidebar). This research shows that ovary removal in adult animals resembles menopause in women and results in decreased bone mass. In women, estrogen deficiency after menopause greatly accelerates bone remodeling and results in a net increase in bone resorption. Inhibitors of bone remodeling, which limit the initiation of new remodeling sites, therefore limit the amount of bone resorption and universally reduce the rate of bone loss and reduce fracture risk. Research shows that postmenopausal women who are moderate drinkers have higher bone mass than abstainers, which could mean that moderate alcohol consumption may have an inhibitory effect on bone remodeling (Turner 2000). It should be emphasized that the “increased bone mass” reported represents a relative improvement. It is unlikely that these women are gaining bone; it is more likely that they are losing bone more slowly than are their peers.

**Action of Estrogen on Bone Remodeling**

Research with laboratory animals has provided most of the current information regarding estrogen’s influence on the growth process of long bones, on the maintenance of cancellous bone mass, and on the architectural and cellular changes in bone. Surgical removal of the ovaries (i.e., ovariectomy) in rats and monkeys results in severely low bone mass (i.e., cancellous osteopenia) in the long bones and vertebrae of rats (Wronski et al. 1989) and in the vertebrae of monkeys (Jerome et al. 1986). Ovariectomy increases the extent of bone surfaces lined by osteoblasts and osteoclasts in rat limbs. At the same time, bone formation rates increase, suggesting that ovariectomy increases the frequency of remodeling*.* Ovariectomy of skeletally mature female rats resembles menopause in that cancellous and cortical bone loss occur because of an abnormally high rate of bone remodeling superimposed on a negative remodeling balance. The elevated remodeling of cancellous bone persists in rats a year or more after ovariectomy. Bone formation is increased in ovariectomized monkeys, suggesting that bone loss in nonhuman primates also is associated with increased bone remodeling (Turner et al. 1994).

In ovariectomized rats, estrogen replacement stabilizes cancellous bone volume by reducing the rate of activation of new bone remodeling sites and reestablishing a neutral or positive balance between bone formation and bone resorption during the remodeling cycle (see part D of the figure) (Turner et al. 1994). Estrogen replacement in postmenopausal women also results in a positive remodeling balance (Turner et al. 1994). Thus, research findings in humans and animals demonstrate that estrogen replacement reduces the overall rate of bone remodeling and improves remodeling balance following estrogen deficiency.

—Russell T. Turner and Jean D. Sibonga

ReferencesJeromeCPKimmelDBMcAlisterJAWeaverDSEffects of ovariectomy on iliac trabecular bone in baboonsCalcified Tissue International392062081986309303210.1007/BF02555119TurnerRTRiggsBLSpelsbergTCSkeletal effects of estrogenEndocrine Reviews152753001994807658210.1210/edrv-15-3-275WronskiTJDannLMHornerSLTime course of vertebral osteopenia in ovariectomized ratsBone102953011989280386610.1016/8756-3282(89)90067-7

In addition to the separate effects that alcohol and estrogen have on bone remodeling, research also suggests that alcohol influences the pathways by which estrogen interacts with various tissues. Chronic male alcoholics develop an assortment of endocrine disorders, including infertility, gonadal atrophy, and feminization, caused in part by elevated production of estrogens and low testosterone levels. Testosterone is converted to estrogen in the peripheral tissues, including bone, by the enzyme aromatase. Alcohol increases the activity of this enzyme (Chung 1990). Thus, it also is possible that higher circulating levels of estrogen account for the higher bone mass reported in women who drink alcohol.

Research investigating the acute and chronic effects of alcohol use on estrogen levels in the blood and urine has had mixed results. One study of post-menopausal women receiving hormone-replacement therapy found that those who drank alcohol had higher blood levels of estrogen compared with those who abstained (Purohit 1998). Increased aromatase activity cannot explain this finding. Instead, it suggests that alcohol slowed the metabolism of estrogen in these women. Not all studies have shown an association between alcohol use and increased estrogen levels (Purohit 1998).

Some research with laboratory animals has suggested that alcohol may help to reduce bone loss by increasing the number of receptors (i.e., binding sites) for estrogen in bone cells (Chung 1990; Fan et al. 2000). Other studies have failed to support this hypothesis, however.

## Summary

Based on limited evidence, the skeletons of women appear to respond differently to alcohol than those of men. In general, the association between heavy alcohol use and decreased bone mass and increased fracture risk is less prevalent in females than in males. Indeed, the weight of evidence suggests that women who consume alcohol generally have a higher bone mass than do abstaining women. There are at least two plausible ways that alcohol could bring about these beneficial effects. Alcohol could enhance estrogenic effects by increasing the circulating levels of the hormone or by increasing the number of estrogen receptors in bone cells. Alternatively, alcohol may act directly on bone cells to inhibit the initiation of bone remodeling, thereby reducing the total number of “weak links” in the skeleton framework. These mechanisms would provide beneficial skeletal effects in older women with elevated bone remodeling rates resulting from menopause. These alternative actions of alcohol are not necessarily mutually exclusive and may be additive.

As with the skeletal effects of estrogen, any beneficial effects of alcohol consumption are context-dependent. Whereas estrogen deficiency-induced bone loss leads to fractures in some women, these problems can be prevented by hormone replacement therapy. The beneficial effects of estrogen replacement must be weighed against the risks. Estrogen replacement has been implicated as a risk factor for breast cancer, another life-threatening disease. The evidence strongly supports the conclusion that alcohol is detrimental to the growing skeleton*.* Furthermore, the many detrimental effects of heavy alcohol use on other organ systems contraindicate any beneficial effect of heavy drinking on the female skeleton. However, because osteoporosis is a life-threatening and debilitating disease with no known cure, even a modest reduction in bone loss could have important positive public health implications. Further research needs to determine if and under what circumstances moderate alcohol consumption may offer some protection against bone loss in aging women.

## Figures and Tables

**Figure:**
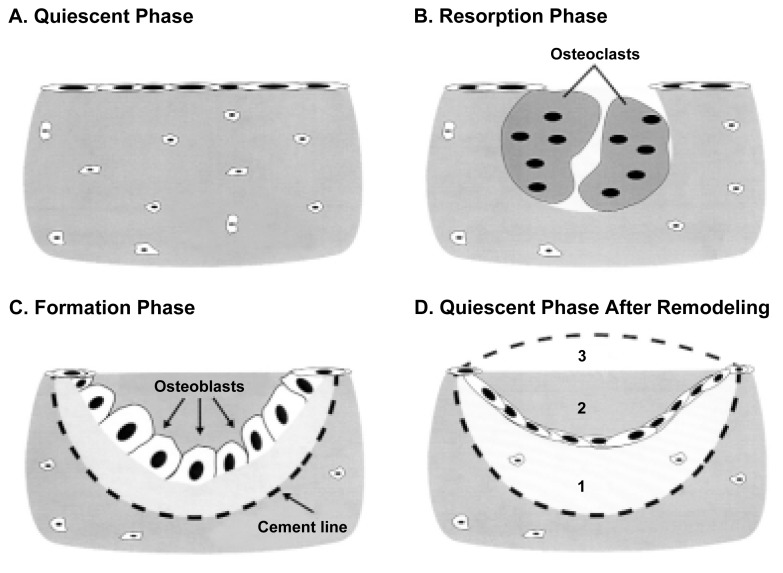
The bone remodeling cycle: Breaking down and rebuilding. Bone is constantly being remodeled by two kinds of cells. Osteoblasts form new bone, while osteoclasts promote resorption, the process by which small packets of bone are broken down and assimilated. These processes permit bone to restructure itself in response to stress and help to maintain strong bones. A. Quiescent phase. Bone surface is inactive. Neither bone resorption nor bone formation is occurring on this region of bone surface. B. Resorption phase. Osteoclasts (i.e., bone-resorbing cells) remove a discrete packet of bone, creating a cavity, a temporary weak spot or link. C. Formation phase. Osteoblasts form a bone matrix, which fills in the cavity. The cement line defines the boundary between the newly formed bone and the surface excavated by osteoclasts at the end of the resorption phase (B). D. Quiescent phase. Inactive bone surface after a completed remodeling cycle. The new surface may be underfilled (1), exactly filled (2), or overfilled (3), reflecting a local decrease, no change, or increase in bone mass, respectively. The most likely mechanism for alcohol-induced bone loss in adults is underfilling of the resorption cavity during bone remodeling.

**Table:** How Alcohol Might Influence Bone Mass in Adolescent, Adult, and Postmenopausal Women

Age	Attribute or Parameter	Effects of Estrogen	Effects of Estrogen and Alcohol
**Adolescent**			
	Bone Growth	↓	↓
	Bone Density	↑	↔
**Young Adult**			
	Remodeling Rate	↓	↓
	Remodeling Balance	↔	↓
	- Formation	↓	↓
	- Resorption	↓	↓
	Density	↔	↓
		**Effects of Estrogen Deficiency**	**Effects of Alcohol**

**Postmenopausal**			
	Remodeling Rate	↑	↔
	Remodeling Balance	↓	↓
	- Formation	↑	↔
	- Resorption	↑	↑
	Density	↓	↓

NOTE: Bone remodeling is the process by which small areas of bone are destroyed and rebuilt. Bone resorption is the first step in bone remodeling, in which a small packet of bone is broken down. Bone formation is the rebuilding process that follows bone resorption. An imbalance in bone remodeling occurs when bone formation does not adequately compensate for bone resorption. Bone loss occurs as a result. Estrogen deficiency accelerates bone remodeling and leads to an imbalance in remodeling. Alcohol might reduce bone loss in postmenopausal women by increasing the circulating levels of estrogen, or by acting on bone cells to reduce bone remodeling. Decreased bone density increases the risk for osteoporosis. Size of arrow represents the magnitude of change.

↑ indicates increase

↓ indicates decrease

↔ indicates no net change

## Footnotes

1 There is no universally accepted definition of heavy alcohol use. The U.S. Government has defined moderate drinking as no more than one drink per day for women and no more than two drinks per day for men. A drink is considered to be 12 ounces (oz) regular beer, 5 oz wine, or 1.5 oz 80-proof distilled spirits (USDHHS and USDA 1995). The risks for detrimental health effects of drinking increase greatly when consumption exceeds these levels.

## References

[b17-arcr-25-4-276] Jerome CP, Kimmel DB, McAlister JA, Weaver DS (1986). Effects of ovariectomy on iliac trabecular bone in baboons. Calcified Tissue International.

[b18-arcr-25-4-276] Turner RT, Riggs BL, Spelsberg TC (1994). Skeletal effects of estrogen. Endocrine Reviews.

[b19-arcr-25-4-276] Wronski TJ, Dann LM, Horner SL (1989). Time course of vertebral osteopenia in ovariectomized rats. Bone.

